# Supplementary material for: Timing of Deep and REM Sleep Based on Fitbit Sleep Staging in Young Healthy Adults under Real-Life Conditions
Source: Brain Sci. 2024 Mar 6;14(3):260. doi: 10.3390/brainsci14030260 (PMC10968898; doi:10.3390/brainsci14030260)
Supplement: Supplementary file 1 [file brainsci-14-00260-s001.zip › Supplementary figure captions.pdf]

Supplemental figure S1. Longitudinal recordings of Fitbit sleep stages. Double-plotted actograms of a repre-sentative subject's sleep recordings. (a) light sleep; (b) deep sleep; (c) REM sleep. Bars indicate sleep stage activity; red dots indicate the respective acrophase.

Supplementary figure S2. Intraindividual timing of sleep stages relative to each other. Heatmaps of the Spearman correlation coefficients of the sleep stage percentiles in (**a**) minutes after sleep onset, and (**b**) clock time of one representative subject. Workdays (WD) and work-free days (FD) were evaluated separately to account for workday constraints, respectively.

Supplementary figure S3. Temporal relationship between deep and REM sleep with regard to the *homeostatic* component. Intraindividual correlation coefficients (ICC) of the deep sleep percentiles and the (**a**) 25 REM sleep percentile, (**b**) 50 REM sleep percentile, (**c**) 75 REM sleep percentile, (**d**) 100 REM sleep percentile. Workdays (green symbols) and work-free days (blue symbols) were evaluated separately to account for workday constraints. Middle lines and error bars show median with interquartile range (N=59). Wilcoxon test, *, P<0.05 between workdays and work-free days. $, P<0.05 vs. the respective 25 percentile.

Supplementary figure S4. Temporal relationship between deep and REM sleep with regard to the *circadian* component. Intraindividual correlation coefficients (ICC) of the deep sleep percentiles and the (**a**) 25 REM sleep percentile, (**b**) 50 REM sleep percentile, (**c**) 75 REM sleep percentile, (**d**) 100 REM sleep percentile. Workdays (green symbols) and work-free days (blue symbols) were evaluated separately. Middle lines and error bars show median with interquartile range (N=59). Wilcoxon test, *,P<0.05 between workdays and work-free days. §, P<0.05 vs. the respective 100 percentile.
