# Supplementary material for: Timing of Deep and REM Sleep Based on Fitbit Sleep Staging in Young Healthy Adults under Real-Life Conditions
Source: Brain Sci. 2024 Mar 6;14(3):260. doi: 10.3390/brainsci14030260 (PMC10968898; doi:10.3390/brainsci14030260)
Supplement: Supplementary file 1 [file brainsci-14-00260-s001.zip › Supplementary figure S2.pdf]

(**a**) **WD**

REM \ Deep	25	50	75	100
25	0.34	0.19	-0.04	-0.06
50	0.34	0.24	-0.06	-0.07
75	0.26	0.26	-0.03	0.15
100	0.35	0.33	0.08	0.17

FD

REM \ Deep	25	50	75	100
25	0.34	0.34	0.33	0.34
50	0.22	0.33	0.33	0.38
75	0.21	0.41	0.32	0.40
100	0.40	0.45	0.46	0.52

(**b**) **WD**

REM \ Deep	25	50	75	100
25	0.60	0.48	0.40	0.20
50	0.60	0.46	0.35	0.16
75	0.54	0.46	0.36	0.31
100	0.38	0.44	0.38	0.32

FD

REM \ Deep	25	50	75	100
25	0.73	0.70	0.68	0.40
50	0.63	0.63	0.55	0.37
75	0.69	0.72	0.55	0.45
100	0.63	0.60	0.52	0.49
