# Supplementary figures and images for: Timing of Deep and REM Sleep Based on Fitbit Sleep Staging in Young Healthy Adults under Real-Life Conditions
Source: Brain Sci. 2024 Mar 6;14(3):260. doi: 10.3390/brainsci14030260 (PMC10968898; doi:10.3390/brainsci14030260)

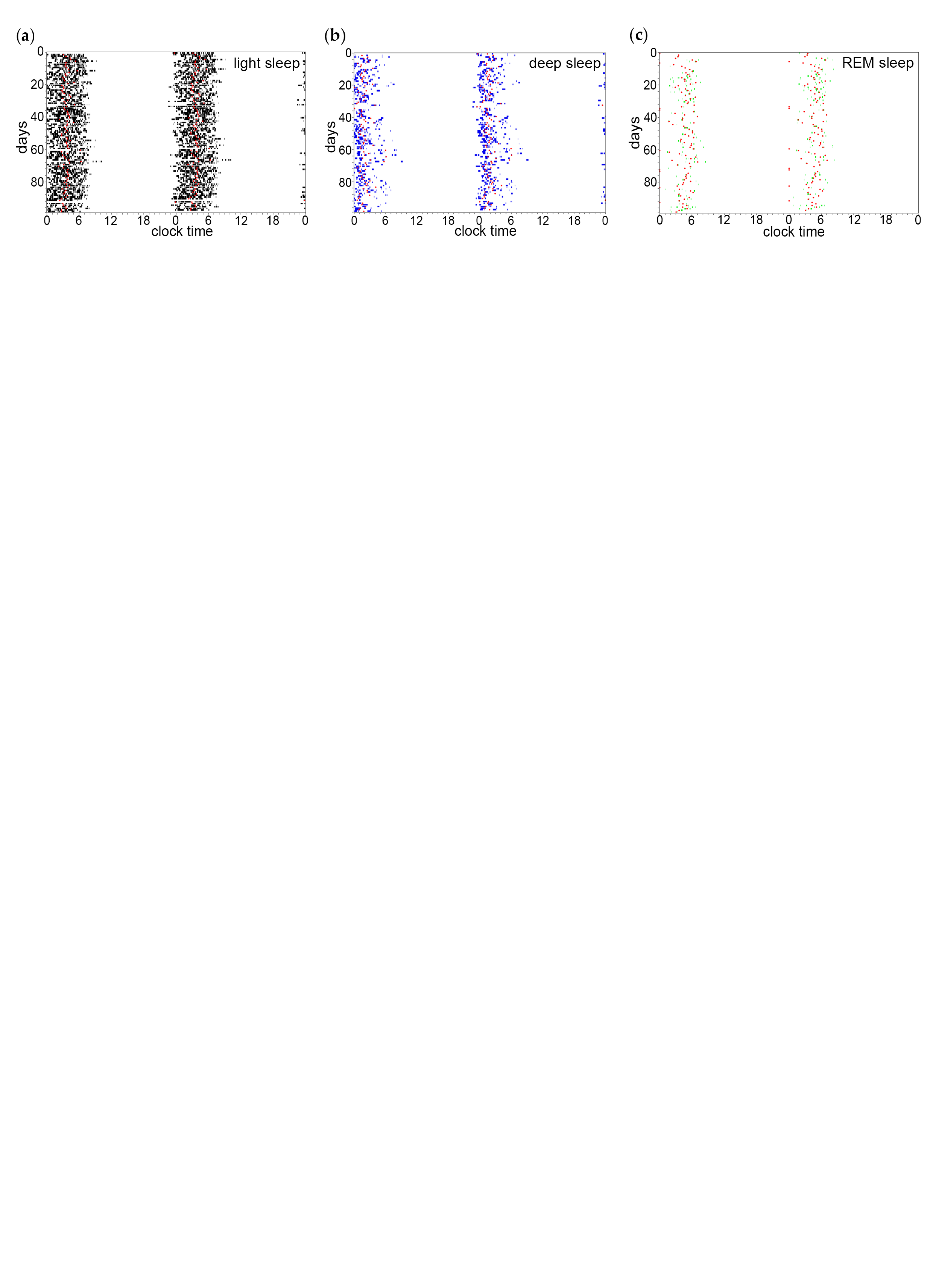

Supplement: Supplementary file 1 [file brainsci-14-00260-s001.zip › Supplementary figure S1.tif]

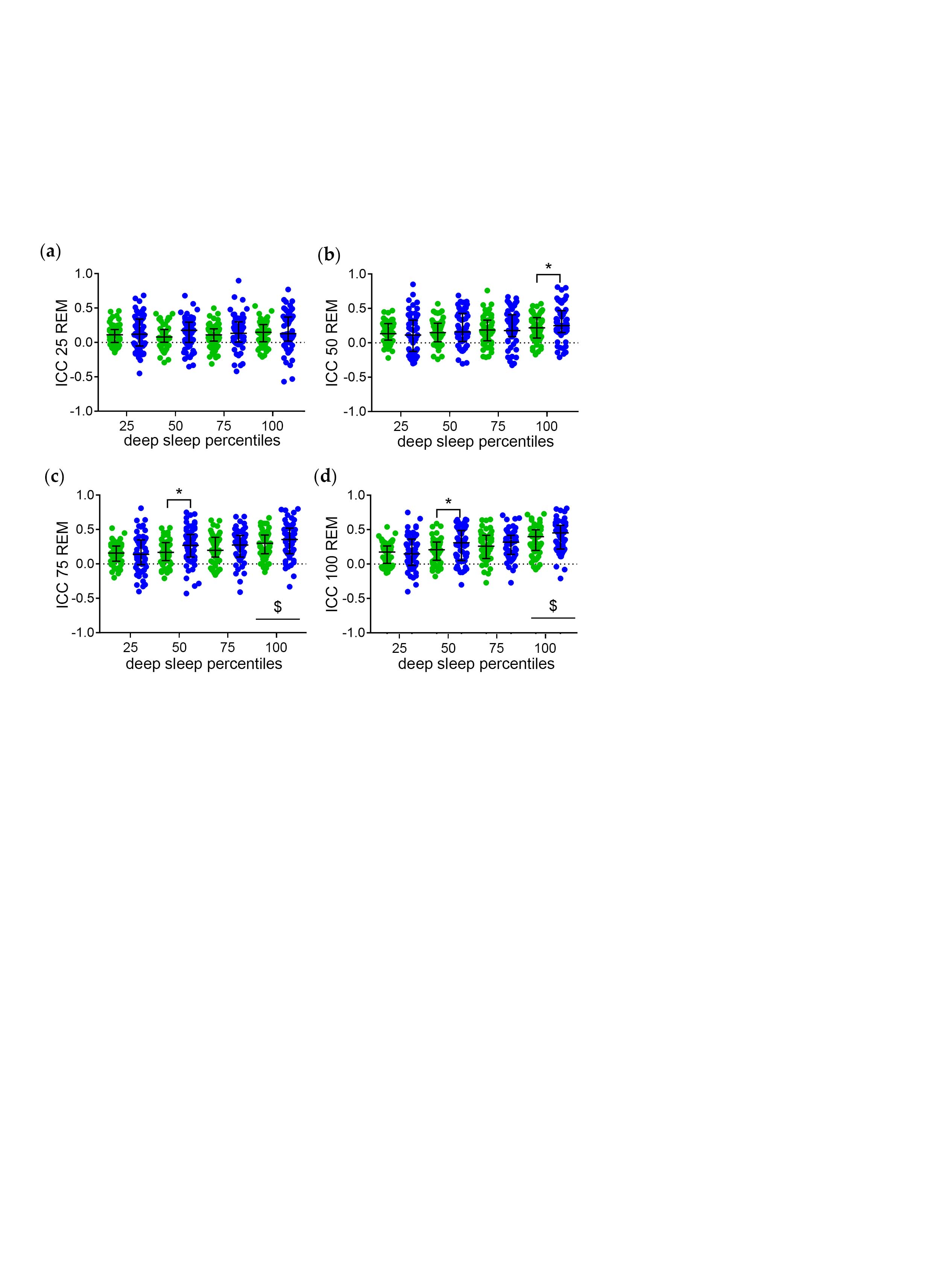

Supplement: Supplementary file 1 [file brainsci-14-00260-s001.zip › Supplementary figure S3.jpg]

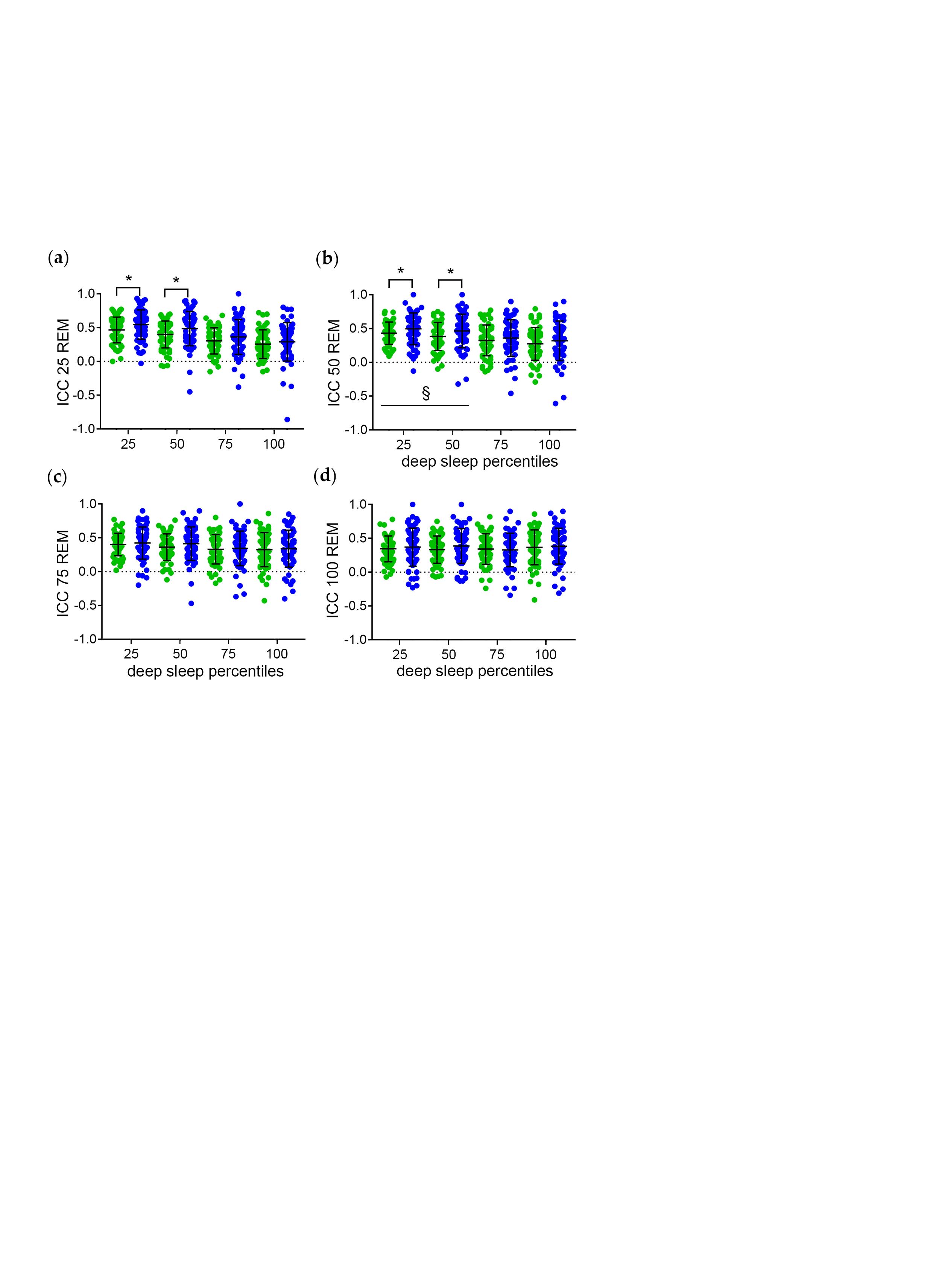

Supplement: Supplementary file 1 [file brainsci-14-00260-s001.zip › Supplementary figure S4.jpg]
